# Machine learning-based prediction of HCV-related cirrhosis and the exploratory analysis of drug use influence

**DOI:** 10.3389/fpubh.2026.1980930

**Published:** 2026-10-13

**Authors:** Shuanglin Gu, Lingling Ye, Jiasheng Tang, Wenxian Qian, Zheng Hu, Xiuchang Ma, Apeng Chen, Qin Zheng, Lei Wang, Guoping Yin, Changhua Yi

**Affiliations:** 1 Department of Clinical Research Center, The Second Hospital of Nanjing, Affiliated to Nanjing University of Chinese Medicine, Nanjing, China; 2 Department of Anesthesiology, The Second Hospital of Nanjing, Affiliated to Nanjing University of Chinese Medicine, Nanjing, China; 3 The Second Hospital of Nanjing, Affiliated to Nanjing University of Chinese Medicine, Nanjing, China; 4 Nanjing Key Laboratory of Pediatrics, Children’s Hospital of Nanjing Medical University, Nanjing, China; 5 Department of Oncology, The Second Hospital of Nanjing, Affiliated to Nanjing University of Chinese Medicine, Nanjing, China; 6 Department of Hepatology, The Second Hospital of Nanjing, Affiliated to Nanjing University of Chinese Medicine, Nanjing, China

**Keywords:** cirrhosis, drug users, hepatitis C virus, machine learning, prediction model

## Abstract

**Introduction:**

Chronic hepatitis C virus (HCV) infection easily progresses to liver cirrhosis, and drug users represent a special high-risk population with accelerated liver damage and insufficient early screening strategies.

**Methods:**

This study aimed to develop a machine learning model for identifying HCV-related cirrhosis, with stratified analysis between drug-using and non-drug-using subgroups. We enrolled patients with chronic HCV infection and collected blood routine, liver function, lymphocyte subsets, and drug use data.

**Results:**

Among the seven models compared, the XGBoost model achieved the best performance, with an AUC of 0.86 on the test set. Stratified analysis showed significantly better predictive performance in drug users (AUC = 0.87) than in non-drug users (AUC = 0.72, *p* < 0.05). Drug use did not independently improve model performance and showed significant interactions with only a limited number of key features (e.g., ADA). SHAP and decision curve analysis validated the model’s reliability and clinical benefit, especially in drug users.

**Discussion:**

The XGBoost model enables noninvasive and accurate cirrhosis prediction and is suitable for early screening in high-risk drug-using populations.

## Introduction

Chronic hepatitis C virus (HCV) infection remains a major global public health concern, with a substantial number of individuals infected worldwide, the majority of whom progress to chronic hepatitis (1). Persistent hepatic inflammation gradually induces liver fibrosis and may ultimately lead to cirrhosis, hepatocellular carcinoma, and even liver failure, posing a serious threat to patient health and imposing a heavy burden on healthcare systems (2, 3). Cirrhosis has an insidious onset with atypical early clinical manifestations. Once decompensation occurs, patients are prone to severe complications such as ascites, hepatic encephalopathy, and gastrointestinal bleeding, resulting in a poor prognosis (4). Therefore, establishing an accurate prediction model to enable early identification and individualized risk stratification of cirrhosis in HCV-infected patients is of great clinical significance for early intervention, delaying disease progression, and improving long-term outcomes (5, 6).

Liver biopsy remains the gold standard for assessing liver fibrosis stage. However, it is an invasive procedure associated with certain risks of complications. In recent years, non-invasive methods for cirrhosis prediction have garnered increasing attention. Transient elastography has become the preferred detection tool, while indices such as the FIB-4 index and the aspartate aminotransferase-to-platelet ratio index (APRI) are also widely used for liver fibrosis assessment (7). Nevertheless, these approaches rely predominantly on single or limited clinical indicators, exhibiting limited predictive performance and failing to meet the demands of precise clinical evaluation (8). Recently, machine learning techniques, with their powerful capacity for multi-feature integration and complex relationship mining, have been widely applied in disease prediction. Compared with traditional statistical models, machine learning methods offer significant improvements in predictive accuracy and model stability, providing a novel technical approach for the early detection of chronic diseases such as cirrhosis (9–11).

Drug use is a major high-risk route for the transmission of hepatitis C. Due to factors such as the sharing of injection equipment and high-risk exposure behaviors, the incidence of HCV infection among drug users is significantly higher than in the general population, making them a key high-risk group for HCV infection (12, 13). At the same time, drugs themselves can directly cause liver cell damage, interfere with liver metabolic function, and disrupt the body’s immune homeostasis, producing a synergistic hepatotoxic effect with HCV infection. This significantly accelerates the progression of liver fibrosis, resulting in a significantly higher risk of cirrhosis and a faster disease progression in this population compared to non-drug-using HCV-infected individuals (14, 15). However, the drug-using population generally faces challenges such as poor follow-up adherence, insufficient standardized screening, and incomplete clinical data collection. To date, there remains a lack of a cirrhosis risk prediction system specifically tailored to HCV-infected drug users, impeding early screening and risk management in this high-risk population (16).

Previous studies have applied machine learning approaches across different levels of HCV care: at the population level, Ding et al. developed and validated a machine learning model using demographic features to screen for prior or current HCV infection in the US population (17). At the disease-progression level, Konerman et al. employed random forest and boosting algorithms to predict fibrosis progression and liver-related outcomes in chronic hepatitis C (18). Together, these studies demonstrate the feasibility of data-driven risk stratification across the HCV care continuum. Building on this foundation, current domestic and international studies have developed several cirrhosis prediction models for chronic hepatitis C populations. These models are mostly constructed based on routine clinical biochemical and hematological parameters, with study populations primarily comprising general hepatitis patients. Few studies have focused on the specific high-risk subgroup of drug users, and the applicability of these models to drug-using populations remains to be validated (19). Several key scientific gaps persist in existing research. First, it remains unclear whether the predictive performance of machine learning models differs between drug-using and non-drug-using HCV subgroups, and whether such models can accurately identify individuals at high risk of cirrhosis among drug users. Second, whether drug use status constitutes an independent influencing factor for cirrhosis, and the extent of its contribution to the overall discriminative ability of the prediction model, have not been quantitatively validated. Third, whether drug use modifies the predictive effect patterns of routine blood parameters, liver function biochemical indicators, and lymphocyte immune subsets on cirrhosis has not been systematically investigated. Moreover, existing studies have predominantly employed a single machine learning model to construct prediction tools, lacking comparative screening across multiple models, making it difficult to identify the best-performing model for the drug-using HCV population. Additionally, some models lack interpretability analyses, failing to elucidate the predictive value and effect patterns of individual features, thereby limiting their clinical translation and application.

This study was conducted based on the above background and existing gaps. We enrolled patients with chronic HCV infection and integrated multidimensional clinical data, including routine blood parameters, liver function biochemical indicators, lymphocyte subsets, and drug use exposure status. Seven machine learning algorithms were employed to construct cirrhosis prediction models, and model selection was achieved through comprehensive performance comparison. This study focuses primarily on drug use stratification. It aims to address the following scientific questions through stratified analysis, feature ablation, interaction testing, and interpretability analysis: (1) whether the predictive performance of the optimal machine learning model for cirrhosis significantly differs between drug-using and non-drug-using HCV populations; (2) whether drug use status has an independent predictive value for cirrhosis and the extent of its contribution to the overall discriminative ability of the optimal model; and (3) whether drug use status modulates the predictive effects of core indicators, including liver function and immune cell subsets, on cirrhosis.

This study conducted systematic modeling, multi-model comparison, and interpretability analysis. The goal was to identify key predictive features and elucidate their effect patterns. These efforts provide reliable model support and a theoretical basis for early screening, risk assessment, and clinical management of cirrhosis in HCV-infected individuals with a history of drug use.

## Materials and methods

### Study population

The study population was derived from patients admitted to the Department of Hepatology at The Second Hospital of Nanjing (Public Health Medical Center). This retrospective study consecutively enrolled patients with HCV infection who were hospitalized between 2021 and 2025. Patients who met the clinical diagnostic criteria for chronic hepatitis C (defined as persistent HCV RNA positivity for at least 6 months) or HCV-related cirrhosis and fulfilled at least one of the following confirmatory criteria were included: (1) positive for both anti-HCV antibody and HCV RNA (detected by real-time quantitative polymerase chain reaction); (2) positive for HCV core antigen; or (3) positive for molecular biology assays (e.g., genotyping or nucleic acid amplification testing [NAAT]). Additionally, patients with histopathological findings of interface hepatitis, portal lymphocyte infiltration, and fibrosis stage ≥ F2 on liver biopsy were diagnosed with chronic hepatitis, while those demonstrating pseudolobule formation and/or regenerative nodules were diagnosed with cirrhosis. Exclusion criteria were as follows: (1) absence of concurrent clinical biochemical (liver function, routine blood tests) and immunological indicator assessments; (2) co-infection with HIV (regardless of positive test result or clinical suspicion); (3) co-infection with other viral hepatitis (hepatitis A, B, or E); (4) presence of severe cardiac, pulmonary, renal, or cerebral dysfunction, or malignancy; (5) prior receipt of antiviral therapy (interferon or direct-acting antivirals [DAAs]); and (6) pregnancy or lactation. These exclusions were intended to minimize confounding from factors that independently affect liver function, hematologic indices, or immune status, thereby allowing the modeled indicators to reflect the natural course of untreated HCV infection in a relatively homogeneous cohort. The study was conducted in strict accordance with the principles of medical ethics and was approved by the Institutional Ethics Committee.

### Statistical analysis

Data were extracted from the hospital information system, integrating patients’ demographic characteristics, laboratory biochemical indicators, and initial admission records. Subject selection strictly adhered to data completeness criteria. Clinically confirmed hepatitis C patients were enrolled and categorized into four groups based on drug use status and disease stage: drug users with chronic hepatitis C (D-Hep), drug users with HCV-related cirrhosis (D-Cirr), non-drug users with chronic hepatitis C (ND-Hep), and non-drug users with HCV-related cirrhosis (ND-Cirr). A total of 22 clinical laboratory indicators were included, comprising 10 T-cell subset parameters (CD3^+^ total T cells, CD3^+^ T cell percentage, CD4^+^/CD45^+^, CD4^+^CD8^+^/CD45^+^, CD4^+^/CD8^+^ T cells, CD4^+^ T cells, CD45^+^ lymphocytes, CD8^+^/CD45^+^, CD8^+^ T cells, and the Th/Ts [CD4^+^/CD8^+^] ratio), 9 liver function parameters (total bilirubin [TBIL], albumin [ALB], direct bilirubin [DBIL], alkaline phosphatase [ALP], gamma-glutamyl transferase [GGT], aspartate aminotransferase [AST], alanine aminotransferase [ALT], cholinesterase [CHE], and adenosine deaminase [ADA]), and 3 routine blood parameters (hematocrit [HCT], platelet count [PLT], and hemoglobin [HGB]). All laboratory measurements were obtained at admission using standard automated assays. Absolute lymphocyte subset counts were expressed in cells/μL, subset percentages in %, and the CD4^+^/CD8^+^ and Th/Ts ratios as unitless ratios. Liver enzymes (ALT, AST, ALP, GGT, CHE, ADA) were reported in U/L; bilirubin (TBIL, DBIL) in μmol/L; albumin in g/L; hematocrit in %; platelet count in ×10^9^/L; and hemoglobin in g/L. The dataset was randomly split into a training set (70%) and a test set (30%) in a 7:3 ratio using stratified random sampling (caret: createDataPartition). The original training set comprised 77 D-Hep, 28 D-Cirr, 185 ND-Hep, and 61 ND-Cirr patients (total *n* = 351), and the test set comprised 32 D-Hep, 11 D-Cirr, 79 ND-Hep, and 25 ND-Cirr patients (total *n* = 147).

Continuous variables with skewed distributions were described using medians and interquartile ranges. Between-group differences were compared using the Kruskal–Wallis test, followed by Dunn’s post-hoc test for pairwise comparisons. To control for the risk of false positives due to multiple comparisons, raw *p*-values were adjusted using the false discovery rate method, implemented via the p.adjust function in R with method = “fdr.” Adjusted *p*-values were used as the threshold for determining statistical significance.

To improve data quality and reduce multicollinearity, optimization was performed on the initial 22 features. Pairwise Pearson correlation coefficients were calculated based on the training set, and features with an absolute correlation coefficient greater than 0.7 were removed using the caret package. The selected features were then standardized using Z-score normalization. Predictive feature selection was subsequently performed using least absolute shrinkage and selection operator regression with the glmnet package (version 4.1.10). Based on the standardized training data, ten-fold cross-validation was used to select the optimal regularization parameter λ that minimized cross-validation error. Features with non-zero coefficients were retained as modeling variables.

To address the underrepresentation of cirrhotic patients in the drug-using group within the training set, the synthetic minority over-sampling technique was applied. Oversampling was performed exclusively on cirrhotic samples in the drug-using group to increase their number to a level comparable to that of the drug-using chronic hepatitis C group. Rather than oversampling the entire cirrhosis class, this subgroup-specific approach was chosen because the most severe class imbalance was concentrated within the drug-using stratum (D-Hep: D-Cirr ≈ 2.8:1 in the full cohort), whereas the non-drug-using cirrhosis subgroup (ND-Cirr, *n* = 86) already had sufficient representation. Oversampling the entire cirrhosis class would have over-represented ND-Cirr and distorted its decision boundary. Non-drug-user samples retained their original distribution, thereby alleviating class imbalance while preserving the distributional characteristics of real-world clinical data. As a sensitivity analysis, class weighting was used as an alternative to SMOTE to assess the robustness of the results (20). SMOTE was selected as the primary approach rather than class weighting because, for the extremely small D-Cirr subgroup (28 cases in the original training set), SMOTE expands minority-class feature-space coverage through k-nearest-neighbor interpolation and increases sample diversity, whereas class weighting merely amplifies the gradients of existing samples and may be more sensitive to outliers. After oversampling, the training set comprised 100 D-Hep, 70 D-Cirr, 185 ND-Hep, and 61 ND-Cirr patients (total *n* = 416), with the D-Hep and D-Cirr subgroups expanded from 77 to 100 and from 28 to 70, respectively, while ND-Hep and ND-Cirr remained unchanged.

### Building diagnostic models and model explanation

Seven machine learning algorithms were employed to develop predictive models for cirrhosis: Random Forest (RF), eXtreme Gradient Boosting (XGBoost), LightGBM, Support Vector Machine (SVM), K-Nearest Neighbor (KNN), Ridge, and GBM (21, 22). Model performance was assessed using a hierarchical evaluation framework. The area under the receiver operating characteristic curve served as the primary metric, supplemented by accuracy, sensitivity, specificity, positive predictive value(PPV), and negative predictive value (NPV), and F1-score. These classification metrics were calculated using the Youden index-optimal threshold (the threshold maximizing the sum of sensitivity and specificity) derived from the ROC curve of each model on the test set. An independent test set, not involved in model training, was reserved solely for final performance evaluation. The DeLong test was applied to compare the area under the receiver operating characteristic curve (AUC) differences between models to determine the statistical significance of model performance (23). The degree of overfitting was quantified by calculating the difference in AUC between the training and test sets. Calibration was assessed using the Brier score (the mean squared difference between predicted probabilities and observed binary outcomes), the calibration slope, and the calibration intercept, the latter two derived from a logistic regression of the observed outcome on the logit-transformed predicted probabilities. A calibration slope of 1 and an intercept of 0 indicate perfect calibration, whereas a slope below 1 indicates overconfidence and a slope above 1 indicates underconfidence; lower Brier scores indicate better probabilistic accuracy. Calibration plots were generated to visualize the agreement between predicted and observed probabilities. XGBoost model was compared with two established clinical fibrosis scores—the Fibrosis-4 index (FIB-4), calculated as (age × AST) / (PLT × √ALT), and the AST-to-Platelet Ratio Index (APRI), calculated as (AST / upper limit of normal) / PLT × 100—as well as a simple logistic regression model using the LASSO-selected features.

To investigate the impact of drug use status on model performance, two parallel models were developed: one incorporating the drug_use variable and one excluding it. The DeLong test was used to compare the AUC of these two models on the test set, assessing whether the drug_use variable independently and significantly contributed to predictive performance. Binary logistic regression was performed to examine interactions between drug use status and key clinical indicators, aiming to determine whether drug use status modifies the direction or magnitude of the effect of these indicators on cirrhosis prediction. Given the number of interaction tests performed, no formal correction for multiple comparisons was applied, interaction results are therefore interpreted as exploratory.

The best-performing XGBoost model was interpreted using the Shapley Additive Explanations method (SHAP), which provides a robust and intuitive ranking of feature contributions based on model predictions. Analysis was implemented using the fastshap package (version 0.1.1) in R. SHAP values were calculated for each feature, and feature importance bar plots along with SHAP dependence plots for the top 8 features were generated to visualize the direction and magnitude of each feature’s contribution to the predictions. Decision curve analysis was performed to evaluate the clinical net benefit of the model. Net benefit was calculated across different threshold probabilities and compared with two extreme strategies: “treat all” and “treat none.” Furthermore, stratified decision curves were plotted for drug users and non-drug users separately to assess the model’s applicability across different clinical subgroups (24).

## Results

### Baseline clinical and demographic characteristics of study population

A total of 498 patients with chronic HCV infection were enrolled (Supplementary Figure S1). Based on drug use status and cirrhosis outcome, they were divided into four subgroups: drug-using hepatitis group (D-Hep, *n* = 109), drug-using cirrhosis group (D-Cirr, *n* = 39), non-drug-using hepatitis group (ND-Hep, *n* = 264), and non-drug-using cirrhosis group (ND-Cirr, *n* = 86). Baseline demographic profiles, peripheral immune cell subsets, liver function indexes, and routine blood indicators were comprehensively compared among the four groups.

In terms of demographic characteristics, male predominance was observed across all groups. The D-Cirr group exhibited the highest male proportion (89.7%), followed by the D-Hep group (76.2%), ND-Hep group (67.8%), and ND-Cirr group (59.3%). Age distribution analysis showed that most patients were aged 40–60 years in all cohorts. The 40–60-year subgroup accounted for 84.4% of D-Hep patients, 87.2% of D-Cirr patients, 78.0% of ND-Hep patients, and 75.6% of ND-Cirr patients. Patients younger than 40 years were rare in all groups, while the proportion of patients older than 60 years was relatively higher in non-drug-using cirrhosis patients (24.4%), suggesting an age-accumulated disease progression pattern in non-drug populations (Table 1).

**Table 1:** Distribution of patients.

		Overall	D-Hep	D-Cirr	ND-Hep	ND-Cirr
Number (%)		498	109 (21.89)	39 (7.83)	264 (53.01)	86 (17.27)
Sex (%)	F	150 (30.12)	26 (23.85)	4 (10.26)	85 (32.20)	35 (40.70)
	M	348 (69.88)	83 (76.15)	35 (89.74)	179 (67.80)	51 (59.30)
Age (%)	<40	28 (5.21)	12 (11.01)	NA	16 (6.06)	NA
	40–60	397 (80.26)	92 (84.40)	34 (87.18)	206 (78.03)	65 (75.58)
	>60	73 (14.53)	5 (4.59)	5 (12.82)	42 (15.91)	21 (24.42)

Regarding T-cell immunophenotypes, absolute counts—but not relative proportions—differed across groups. Cirrhotic patients had significantly lower absolute counts of CD3^+^ T cells, CD4^+^ T cells, CD8^+^ T cells, and CD45^+^ lymphocytes than patients with hepatitis, with the lowest values observed in the D-Cirr group (e.g., CD3^+^ T: D-Cirr 1,340 vs. D-Hep 1,686, *p* < 0.001; vs. ND-Hep 1529.5, *p* = 0.031). CD8^+^ T cells distinguished cirrhosis from hepatitis mainly among drug users (D-Cirr vs. D-Hep, *p* < 0.001) and in the non-drug group (ND-Cirr vs. ND-Hep, *p* < 0.001), without a significant difference between D-Cirr and ND-Hep (*p* = 0.109). By contrast, the relative proportions of CD4^+^/CD45^+^, CD4^+^/CD8^+^ T cells, and the Th/Ts ratio were comparable across all four groups (all *p* > 0.05), although the CD4^+^CD8^+^/CD45^+^ fraction was significantly lower in D-Hep than in both non-drug groups (both *p* < 0.05).

For laboratory parameters, markers of hepatic dysfunction and portal hypertension differed primarily between cirrhosis and hepatitis rather than by drug-use status. ADA levels were significantly elevated and platelet counts significantly reduced in both cirrhotic groups compared with both hepatitis groups (all *p* < 0.01), with no significant difference between D-Cirr and ND-Cirr (ADA, *p* = 0.294; PLT, *p* = 0.944), indicating that these alterations reflect cirrhosis rather than drug exposure. Similarly, TBIL and ALP were significantly higher in D-Cirr than in both hepatitis groups, and ND-Cirr showed the lowest CHE, HCT, and HGB values among the four groups. Notably, the conventional liver enzymes ALT, GGT, and AST did not differ significantly among the four groups (all *p* > 0.05), underscoring that immunologic and non-enzymatic markers, particularly ADA, PLT, and absolute T-cell counts, better captured the differences between cirrhosis and hepatitis across drug-use strata (Tables 2, 3).

**Table 2:** Baseline demographic characteristics of the study population.

Variable	Overall	D-Hep	D-Cirr	ND-Hep	ND-Cirr
CD3^+^ T%, M (Q_1_, Q_3_)	76.1 (70.3–78.5)	76.1 (75.9–76.3)	76.7 (73.5–78.1)	75.1 (69.7–78.8)	72.6 (66.2–79.2)
CD3^+^ T, M (Q_1_, Q_3_)	1,562.5 (1,200.3–1,844.3)	1,686.0 (1,673.0–1,709.0)	1,340.0 (1,230.5–1,584.5)	1,529.5 (1,198.5–1,983.3)	1,245.0 (766.0–1,726.3)
CD4^+^/CD45^+^, M (Q_1_, Q_3_)	43.0 (39.0–49.0)	43.0 (43.0–43.0)	45.0 (39.5–49.0)	44.0 (38.0–49.3)	44.0 (37.3–48.0)
CD4^+^ CD8^+^/CD45^+^, M (Q_1_, Q_3_)	0.2 (0.1–0.4)	0.2 (0.2–0.2)	0.2 (0.1–0.4)	0.3 (0.1–0.4)	0.2 (0.1–0.4)
CD4^+^/CD8^+^ T, M (Q_1_, Q_3_)	4.0 (2.0–8.0)	4.0 (4.0–4.0)	3.0 (2.0–8.0)	4.0 (2.0–9.0)	4.0 (2.0–8.8)
CD4^+^ T, M (Q_1_, Q_3_)	909.5 (678.3–1,081.0)	948.5 (911.0–962.0)	809.0 (631.0–953.5)	883.5 (684.3–1,162.8)	738.5 (438.3–1,024.5)
CD45^+^ _lymph, M (Q_1_, Q_3_)	2,086.0 (1,646.3–2,469.3)	2,217.5 (2,134.0–2,318.0)	1,895.0 (1,711.5–2,047.0)	2,063.0 (1,656.8–2,607.0)	1,673.5 (1,115.8–2,232.0)
CD8^+^/CD45^+^, M (Q_1_, Q_3_)	26.5 (21.0–30.0)	27.0 (27.0–27.0)	26.0 (22.0–29.0)	26.0 (20.3–31.0)	24.0 (19.9–28.8)
CD8^+^ T, M (Q_1_, Q_3_)	546.0 (366.0–655.5)	605.5 (568.0–621.0)	478.0 (349.5–573.5)	529.0 (377.0–679.5)	367.0 (270.3–625.5)
Th/Ts(CD4^+^/CD8^+^), M (Q_1_, Q_3_)	1.7 (1.4–2.2)	1.7 (1.7–1.7)	1.8 (1.5–2.1)	1.7 (1.3–2.3)	1.8 (1.4–2.4)
TBIL, M (Q_1_, Q_3_)	13.0 (9.6–17.6)	12.0 (9.0–16.1)	16.0 (10.7–23.4)	12.9 (9.2–16.9)	15.4 (11.4–23.2)
ALB, M (Q_1_, Q_3_)	42.0 (39.5–44.8)	42.9 (40.9–45.9)	42.0 (38.7–44.3)	42.0 (39.7–44.4)	41.0 (37.6–44.0)
DBIL, M (Q_1_, Q_3_)	4.4 (3.0–6.2)	4.6 (3.3–6.1)	5.2 (3.4–10.1)	4.0 (2.9–5.7)	5.6 (3.0–8.3)
ALP, M (Q_1_, Q_3_)	66.0 (54.0–87.0)	65.0 (54.0–84.0)	82.0 (62.5–101.5)	65.0 (53.0–85.0)	70.0 (55.3–98.8)
HCT, M (Q_1_, Q_3_)	42.1 (38.6–45.4)	43.2 (40.5–45.5)	41.8 (39.2–46.3)	42.0 (38.7–45.7)	40.2 (35.4–43.6)
CHE, M (Q_1_, Q_3_)	7,878.5 (6,489.0–8,772.8)	7,942.5 (6,836.0–8,663.0)	7,374.0 (5,802.0–8,452.0)	7,895.5 (6,984.5–8,949.3)	6,552.0 (4,405.3–8,483.8)
ADA, M (Q_1_, Q_3_)	18.9 (14.0–25.9)	15.5 (11.0–22.0)	23.4 (18.8–33.5)	18.9 (14.0–25.4)	22.5 (18.0–28.0)
PLT, M (Q_1_, Q_3_)	159.0 (115.0–204.0)	185.0 (141.0–226.0)	105.0 (77.5–176.0)	164.0 (128.8–206.0)	102.0 (67.3–163.3)
HGB, M (Q_1_, Q_3_)	143.5 (130.0–155.0)	148.5 (140.0–155.0)	142.5 (132.0–155.5)	143.0 (131.0–157.0)	135.0 (117.3–148.8)
ALT, M (Q_1_, Q_3_)	31.2 (15.6–63.3)	36.4 (17.7–64.2)	40.7 (16.5–71.5)	32.3 (15.5–66.6)	25.1 (16.6–44.1)
GGT, M (Q_1_, Q_3_)	41.8 (23.2–87.9)	39.8 (21.1–88.0)	54.0 (30.9–184.8)	41.8 (23.9–84.5)	38.3 (21.5–81.7)
AST, M (Q_1_, Q_3_)	30.0 (20.4–52.8)	34.0 (20.0–54.0)	37.2 (22.9–66.4)	30.0 (20.4–52.5)	27.6 (21.4–49.5)

**Table 3:** Adjusted *p*-values for pairwise comparisons of laboratory parameters among the four groups.

Variable	D-Cirr vs. D-Hep	D-Cirr vs. ND-Cirr	D-Hep vs. ND-Cirr	D-Cirr vs. ND-Hep	D-Hep vs. ND-Hep	ND-Cirr vs. ND-Hep
CD3^+^ T%, M (Q_1_, Q_3_)	0.6635	0.0485*	0.0474*	0.1726	0.2047	0.1709
CD3^+^ T, M (Q_1_, Q_3_)	4e−04***	0.6082	<0.001***	0.0309*	0.0084**	3e−04***
CD4^+^/CD45^+^, M (Q_1_, Q_3_)	1	0.5853	0.7343	0.6077	0.6753	0.709
CD4^+^ CD8^+^/CD45^+^, M (Q_1_, Q_3_)	0.301	0.5989	0.0342*	0.8005	0.0186*	0.7243
CD4^+^/CD8^+^ T, M (Q_1_, Q_3_)	0.5587	0.68	0.9525	0.7138	0.7464	0.646
CD4^+^ T, M (Q_1_, Q_3_)	0.008**	0.6607	1e−04***	0.0527	0.147	0.001***
CD45^+^ _lymph, M (Q_1_, Q_3_)	5e−04***	0.5899	<0.001***	0.0225*	0.0196*	1e−04***
CD8^+^/CD45^+^, M (Q_1_, Q_3_)	0.3472	0.3722	0.0212*	0.8602	0.1358	0.2411
CD8^+^ T, M (Q_1_, Q_3_)	8e−04***	0.3493	<0.001***	0.1087	0.0018**	4e−04***
Th/Ts(CD4^+^/CD8^+^), M (Q_1_, Q_3_)	0.5614	0.929	0.779	0.8054	0.7778	0.7025
TBIL, M (Q_1_, Q_3_)	0.0125*	0.9158	0.0014**	0.0255*	0.453	0.0016**
ALB, M (Q_1_, Q_3_)	0.0404*	0.4346	3e−04***	0.4674	0.0341*	0.031*
DBIL, M (Q_1_, Q_3_)	0.248	0.7979	0.0962	0.0511	0.1909	0.0013**
ALP, M (Q_1_, Q_3_)	0.0236*	0.1886	0.2768	0.0196*	0.9395	0.2156
HCT, M (Q_1_, Q_3_)	0.6019	0.0221*	1e−04***	0.8155	0.2194	9e−04***
CHE, M (Q_1_, Q_3_)	0.0843	0.3197	2e−04***	0.0512	0.8144	<0.001***
ADA, M (Q_1_, Q_3_)	<0.001***	0.2941	<0.001***	0.0021**	0.0014**	0.0067**
PLT, M (Q_1_, Q_3_)	<0.001***	0.9441	<0.001***	1e−04***	0.0548	<0.001***
HGB, M (Q_1_, Q_3_)	0.4904	0.0241*	1e−04***	0.9944	0.2586	3e−04***
ALT, M (Q_1_, Q_3_)	0.561	0.7301	0.5155	0.5994	0.9374	0.6419
GGT, M (Q_1_, Q_3_)	0.0513	0.0906	0.8672	0.0506	0.7092	0.7382
AST, M (Q_1_, Q_3_)	0.4393	0.8031	0.8984	0.3136	0.9662	1

### Feature engineering, model development and performance evaluation

To establish a robust and stable prediction model for cirrhosis classification, a systematic feature selection procedure was performed on 22 clinical laboratory indicators. The workflow included correlation analysis and LASSO regression to eliminate multicollinearity and identify key predictive features. First, Pearson correlation analysis was applied to remove redundant highly correlated features. Variables with an absolute correlation coefficient > 0.7 were excluded, resulting in the removal of CD3^+^ T, CD4^+^ _CD8^+^ _CD45^+^, CD4^+^ T, Th_Ts (CD4^+^/CD8^+^), DBIL and HCT (Figure 1). Subsequently, 10-fold cross-validated LASSO regression (*α* = 1) was used to select features with non-zero coefficients based on the optimal lambda value (Figure 2). Finally, 9 core predictive features were retained, including: CD3^+^ _T%, CD8^+^ _CD45^+^, CD8^+^ _T, TBIL, ADA, HGB, AST, PLT, and ALT. After systematic feature selection, the remaining indicators were free of significant multicollinearity and were used for subsequent model construction and validation using seven machine learning algorithms.

**Figure 1:**
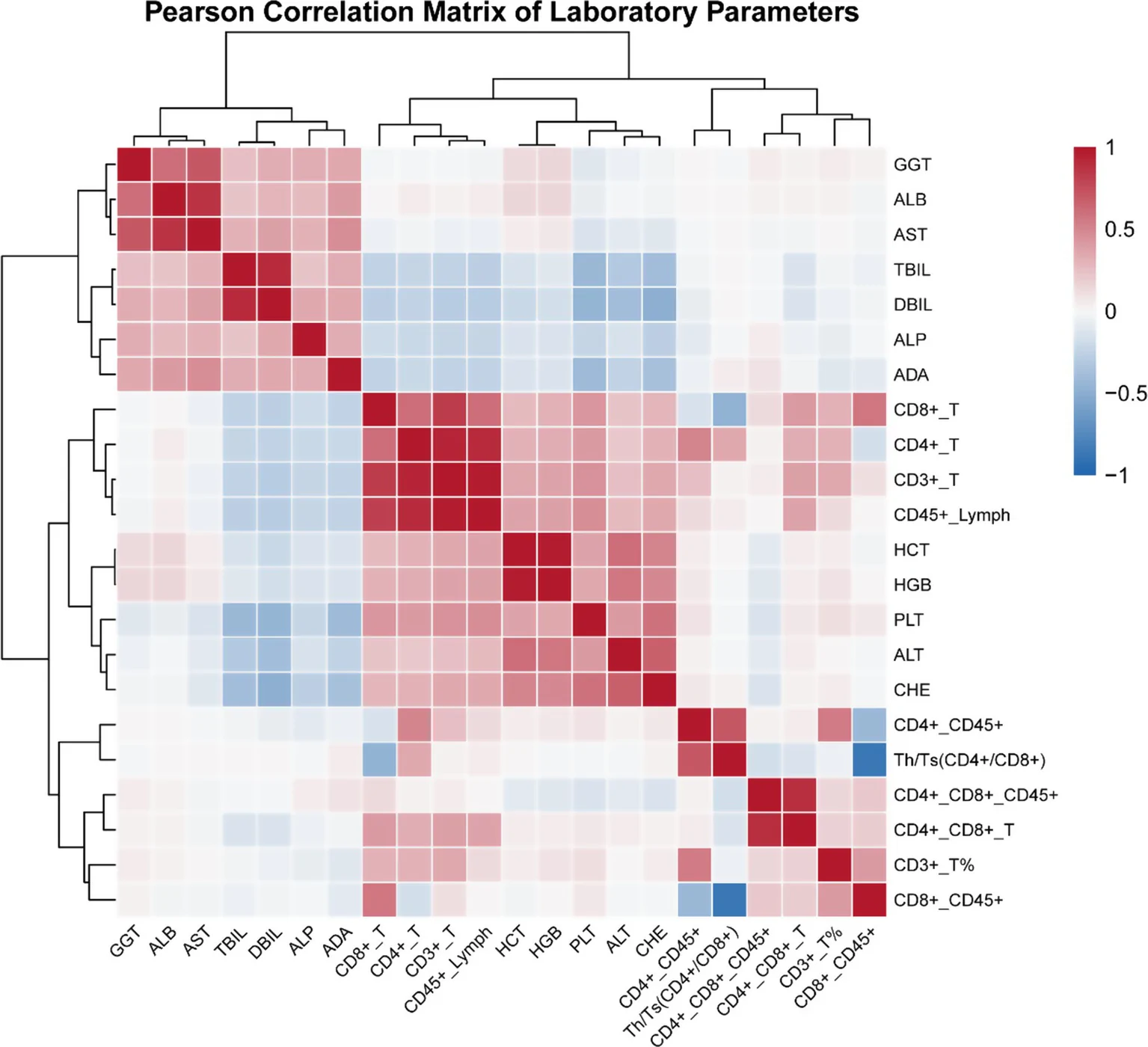
The pairwise correlations between characterization factors were assessed using Pearson’s method. Positive and negative correlations are shown in red and blue, respectively, with greater color intensity (dark blue or dark red) reflecting stronger associations.

**Figure 2:**
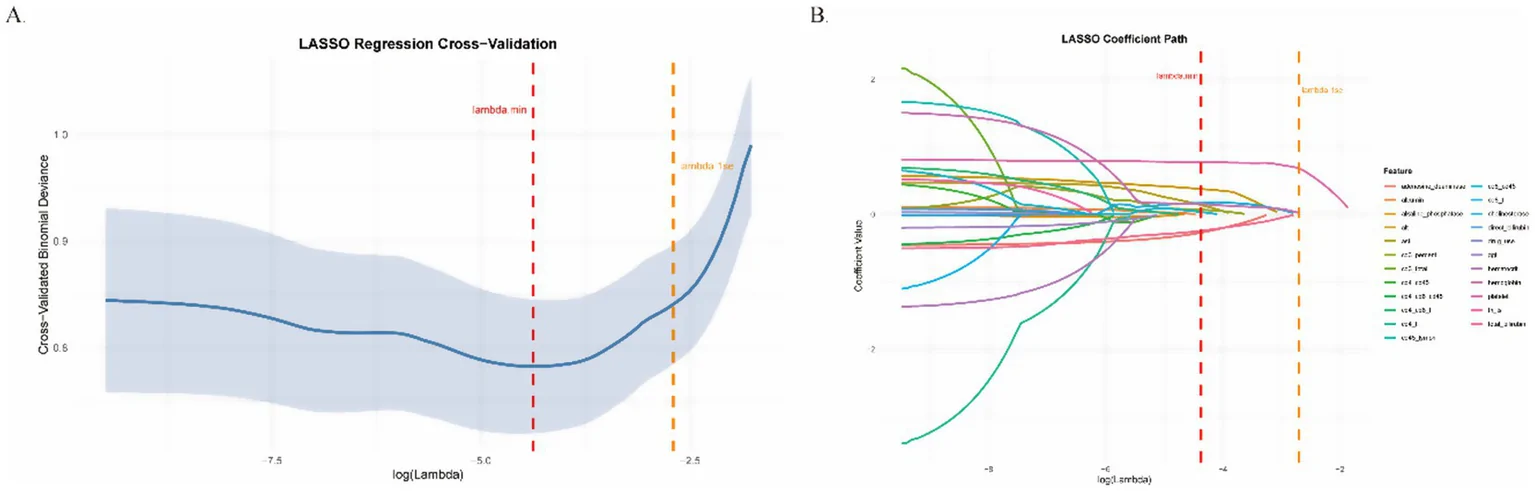
**(A)** LASSO regression cross-validation for selection of the optimal regularization parameter. **(B)** Distribution of coefficients for LASSO (binomial) regression.

Given the cross-sectional study design, the models were developed to predict the presence of prevalent cirrhosis at the time of evaluation rather than the future risk of developing cirrhosis. Based on the 9 features screened by LASSO regression, seven machine learning classification models were constructed to comprehensively compare the predictive performance of different algorithms for HCV-associated liver cirrhosis in the classification task. The models included RF, XGBoost, LightGBM, SVM, KNN, Ridge, and GBM. All models were implemented using the caret package in R software. Parameter optimization and model fitting were performed on the training set, followed by validation on an independent test set. The overall performance of each model was comprehensively evaluated based on criteria such as discriminatory power, predictive accuracy, and generalization performance. Additionally, the DeLong test was used to statistically validate the differences in AUC scores across the test sets for each model, ensuring the reliability of the model selection results.

All models exhibited satisfactory fitting performance in the training set, with the AUC ranging from 0.83 to 0.95 and accuracy ranging from 0.81 to 0.90. Among them, the XGBoost model achieved the best performance in the training set. This was followed by the KNN model (training AUC = 0.93, 95% CI: 0.90–0.95) and the Random Forest model (training AUC = 0.90, 95% CI: 0.86–0.94), both of which showed good fitting effects. The GBM model had the lowest training AUC (0.83, 95% CI: 0.77–0.89), indicating relatively weak fitting ability. Conversely, the KNN model showed the lowest test AUC (0.77, 95% CI: 0.67–0.87) and the poorest generalization capacity despite its high training AUC (0.93), indicating a marked overfitting tendency. The DeLong test further confirmed that the test AUC of the XGBoost model was significantly higher than that of the KNN model (*p* = 0.01), whereas no significant differences were detected between XGBoost and the remaining models (*p* > 0.05).

Regarding key clinical performance metrics, the XGBoost model achieved the most balanced discrimination in the test set, with a sensitivity of 0.86 and a specificity of 0.85, together with the highest F1-score (0.69) and AUPRC (0.96), indicating superior overall ability to identify cirrhosis-positive cases while minimizing false positives. Although the Random Forest model reached the highest test sensitivity (1.00), it did so at the expense of the lowest specificity (0.58) and PPV (0.35), suggesting a tendency toward over-detection of positive cases. For specificity, the KNN and LightGBM models performed best, reaching 0.87 and 0.86 in the test set, respectively, followed by the XGBoost model (0.85) (Table 4, 5; Figure 3).

**Table 4:** Performance of seven machine learning models in predicting HCV-related cirrhosis.

Model	Dataset	AUC (95% CI)	Sensitivity	Specificity	PPV	NPV	Accuracy	F1
XGBoost	Test	0.8604 (0.79–0.93)	0.8571	0.85	0.5714	0.9623	0.8514	0.6857
Random Forest	Test	0.856 (0.79–0.92)	1	0.575	0.3544	1	0.6554	0.5234
LightGBM	Test	0.8491(0.77–0.93)	0.7857	0.8583	0.5641	0.945	0.8446	0.6567
Ridge	Test	0.814(0.73–0.90)	0.8214	0.6833	0.377	0.9425	0.7095	0.5169
GBM	Test	0.8068 (0.71–0.90)	0.7143	0.8167	0.4762	0.9245	0.7973	0.5714
SVM	Test	0.7926 (0.69–0.89)	0.8214	0.725	0.4107	0.9457	0.7432	0.5476
KNN	Test	0.7685 (0.67–0.87)	0.6071	0.8667	0.5152	0.9043	0.8176	0.5574
XGBoost	Train	0.9533 (0.93–0.98)	0.8824	0.9007	0.6818	0.9695	0.8971	0.7692
KNN	Train	0.9272 (0.90–0.95)	0.9559	0.8014	0.5372	0.9869	0.8314	0.6878
Random forest	Train	0.9019 (0.86–0.94)	0.8088	0.8475	0.5612	0.9484	0.84	0.6627
Ridge	Train	0.8413 (0.79–0.89)	0.7059	0.8298	0.5	0.9213	0.8057	0.5854
SVM	Train	0.8361 (0.78–0.89)	0.6618	0.8759	0.5625	0.9148	0.8343	0.6081
LightGBM	Train	0.8325 (0.78–0.89)	0.7353	0.8298	0.5102	0.9286	0.8114	0.6024
GBM	Train	0.8307 (0.77–0.89)	0.6765	0.8723	0.561	0.9179	0.8343	0.6133

PPV, Positive Predictive Value; NPV, Negative Predictive Value; Acc, Accuracy; AUC, Area Under the Curve; CI, Confidence Interval.

**Table 5:** Pairwise comparison of AUC values between different machine learning models using DeLong test.

Model	LightGBM	XGBoost	Random forest	SVM	Ridge	GBM	KNN
LightGBM	–	0.7023	0.8456	0.3169	0.4146	0.2645	0.0838
XGBoost	0.7023	–	0.8668	0.0837	0.1626	0.2172	0.0106
Random forest	0.8456	0.8668	–	0.1344	0.1929	0.1567	0.0161
SVM	0.3169	0.0837	0.1344	–	0.3446	0.809	0.5547
Ridge	0.4146	0.1626	0.1929	0.3446	–	0.8696	0.162
GBM	0.2645	0.2172	0.1567	0.809	0.8696	–	0.3541
KNN	0.0838	0.0106	0.0161	0.5547	0.162	0.3541	–

**Figure 3:**
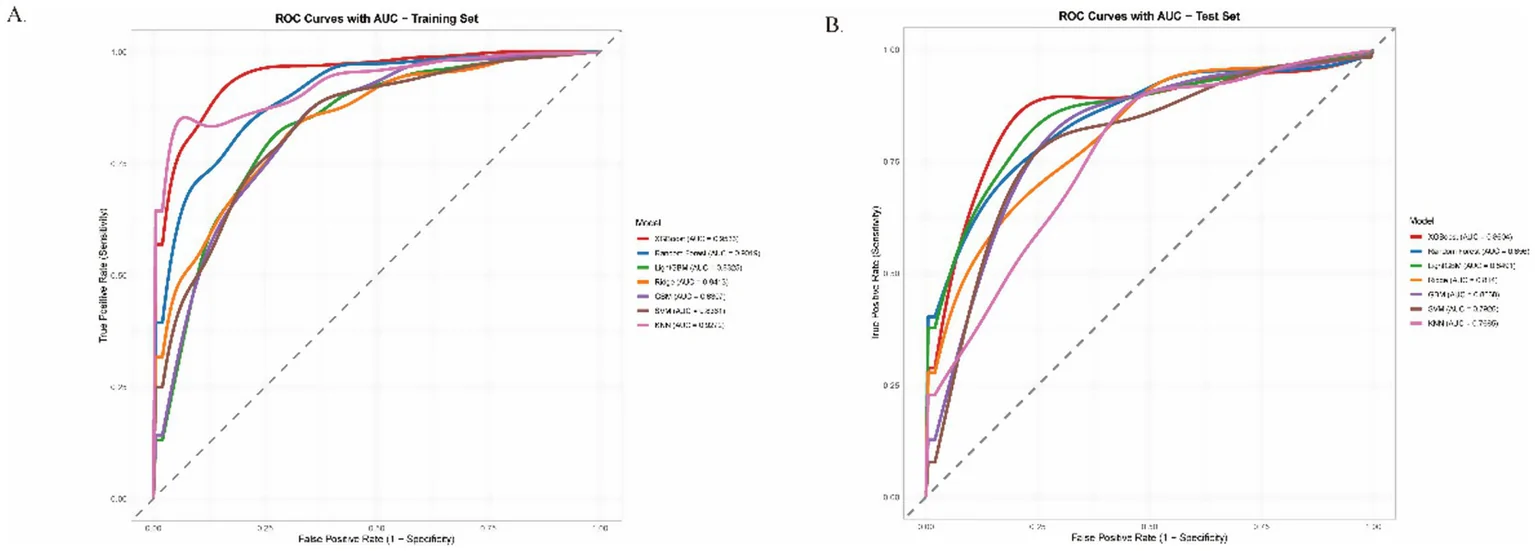
Performance evaluation of the seven machine learning models. ROC-AUC curves are displayed for the **(A)** training set, **(B)** test set.

Based on comprehensive evaluation of training fitting performance, test generalization ability, key clinical metrics, and statistical significance from DeLong testing, XGBoost was identified as the best-performing model for HCV-related cirrhosis prediction in this study. This model achieved the highest test AUC (0.86) among all models, with the most balanced sensitivity (0.86) and specificity (0.85) at the Youden index-optimal probability threshold of 0.216, as well as the highest F1-score (0.69) and AUPRC (0.96) in the test set, while maintaining favorable training AUC (0.95), accuracy (0.90), and F1 score (0.77) (Supplementary Table S3). It balanced goodness-of-fit and generalization ability while effectively avoiding overfitting. DeLong testing further confirmed that the test AUC of XGBoost was significantly superior to that of the KNN model (*p* = 0.01). Thus, XGBoost was selected as the best-performing model based on a comprehensive evaluation of multiple metrics, including the highest test AUC (0.8604), the highest F1-score (0.69) and AUPRC (0.96), the most balanced sensitivity (0.86) and specificity (0.85), the best calibration (Brier score = 0.1238), favorable training–test generalization (AUC gap = 0.09), and built-in feature interpretability, rather than on statistically significant AUC superiority alone. Furthermore, as a gradient-boosting ensemble learning model, XGBoost provides reliable interpretability of feature importance, allowing quantitative assessment of the contribution of each clinical indicator to cirrhosis prediction. To assess whether the model performance was dependent on the specific imbalance-handling method, a sensitivity analysis was performed using class weighting as an alternative to SMOTE. Under class weighting, XGBoost retained a test AUC of 0.8501, which was nearly identical to the SMOTE-based primary result (0.8604), and showed no overfitting. The drug-use subgroup advantage was also preserved, with higher AUROC in drug users (0.8661) than in non-drug users (0.8271), directionally consistent with the primary analysis. These findings indicate that the main conclusions are robust to the choice of imbalance-handling approach (Supplementary Table S1).

Calibration analysis was further conducted to assess the agreement between predicted probabilities and observed event rates. The XGBoost model exhibited the best calibration among all seven models, achieving the lowest Brier score (0.1238), a calibration slope closest to unity (1.181), and a calibration intercept nearest to zero (0.005), indicating that its predicted probabilities closely approximated the observed cirrhosis rates. In contrast, Random Forest showed overconfidence (calibration slope = 0.541), whereas Ridge showed underconfidence (slope = 1.807). LightGBM and GBM displayed poor probability calibration despite acceptable discrimination. The calibration plot for XGBoost is provided in Supplementary Figure S2, Table S2.

To further contextualize the model performance, XGBoost was compared with two established clinical fibrosis scores (FIB-4 and APRI) and a simple logistic regression model. XGBoost achieved the highest test AUC (0.8604; 95% CI: 0.7885–0.9323), followed by logistic regression (0.8003; 95% CI: 0.7021–0.8985), FIB-4 (0.7938; 95% CI: 0.6978–0.8897), and APRI (0.7333; 95% CI: 0.6215–0.8451) (Supplementary Figure S3).

### Impact of drug use status on model predictive performance

To investigate the impact of drug use status on the predictive performance of the XGBoost model, we performed stratified analysis, feature importance analysis, and interaction analysis with a focus on drug use subgroups. Model performance was then compared between drug-using and non-drug-using cohorts (Table 6). Stratified analysis showed that the XGBoost model achieved significantly higher predictive performance in the drug-using population (AUC = 0.87) than in the non-drug-using population (AUC = 0.72, *p* = 0.04), indicating that liver disease in drug users was more distinguishable using routine clinical indicators. However, after removing the drug_use feature, the model AUC was 0.71, which showed no significant difference compared with the model including this feature (0.73, *p* = 0.27) (Figure 4), noting that a non-significant change in AUROC does not rule out an independent association between drug use and cirrhosis, as AUROC may be insensitive to the incremental effect of a single feature. In addition, among all interaction terms, only ADA × drug use reached statistical significance (*p* = 0.03, treated as exploratory given the number of interaction tests performed), whereas the remaining interactions were not significant (all *p* > 0.05). These analyses are exploratory and do not constitute mediation analysis, we therefore avoid drawing causal conclusions about the pathway by which drug use may influence cirrhosis. These findings suggest that routine clinical indicators capture much of the information associated with drug use status, although the cross-sectional design precludes causal inference regarding the pathway by which drug use may influence cirrhosis. In summary, the XGBoost model shows significantly better predictive performance in drug-using patients than in non-drug-using patients. However, the drug use feature itself does not improve overall model performance, nor does it alter the effect pattern of core clinical indicators.

**Table 6:** Stratified analysis, feature importance analysis and interaction effects of drug use status on model performance.

Analysis	Comparison/term	Value	*p*-value	Outcome/interpretation
Stratified analysis	Drug users	0.8665 (0.7472–0.9857)	–	AUC (95% CI)
	Non-drug users	0.7200 (0.6400–0.8000)	–	AUC (95% CI)
	Drug users vs. non-drug users	–	0.0354	Significant
Feature contribution	With drug_use	0.7306 (0.6253–0.8359)	–	AUC (95% CI)
	Without drug_use	0.7102 (0.6023–0.8182)	–	AUC (95% CI)
	With vs. without drug_use	–	0.2696	No significant contribution
Interaction analysis	PLT × drug_use	–	0.4894	No significant
	TBIL × drug_use	–	0.2923	No significant
	ALT × drug_use	–	0.7987	No significant
	CD8^+^ T cell × drug_use	–	0.7937	No significant
	HGB × drug_use	–	0.4888	No significant
	ADA × drug_use	–	0.0302	Significant
	CD3^+^ T% × drug_use	–	0.1089	No significant
	AST × drug_use	–	0.9143	No significant

* *P*.adj < 0.05, ** *P*.adj < 0.01, *** *P*.adj < 0.001.

**Figure 4:**
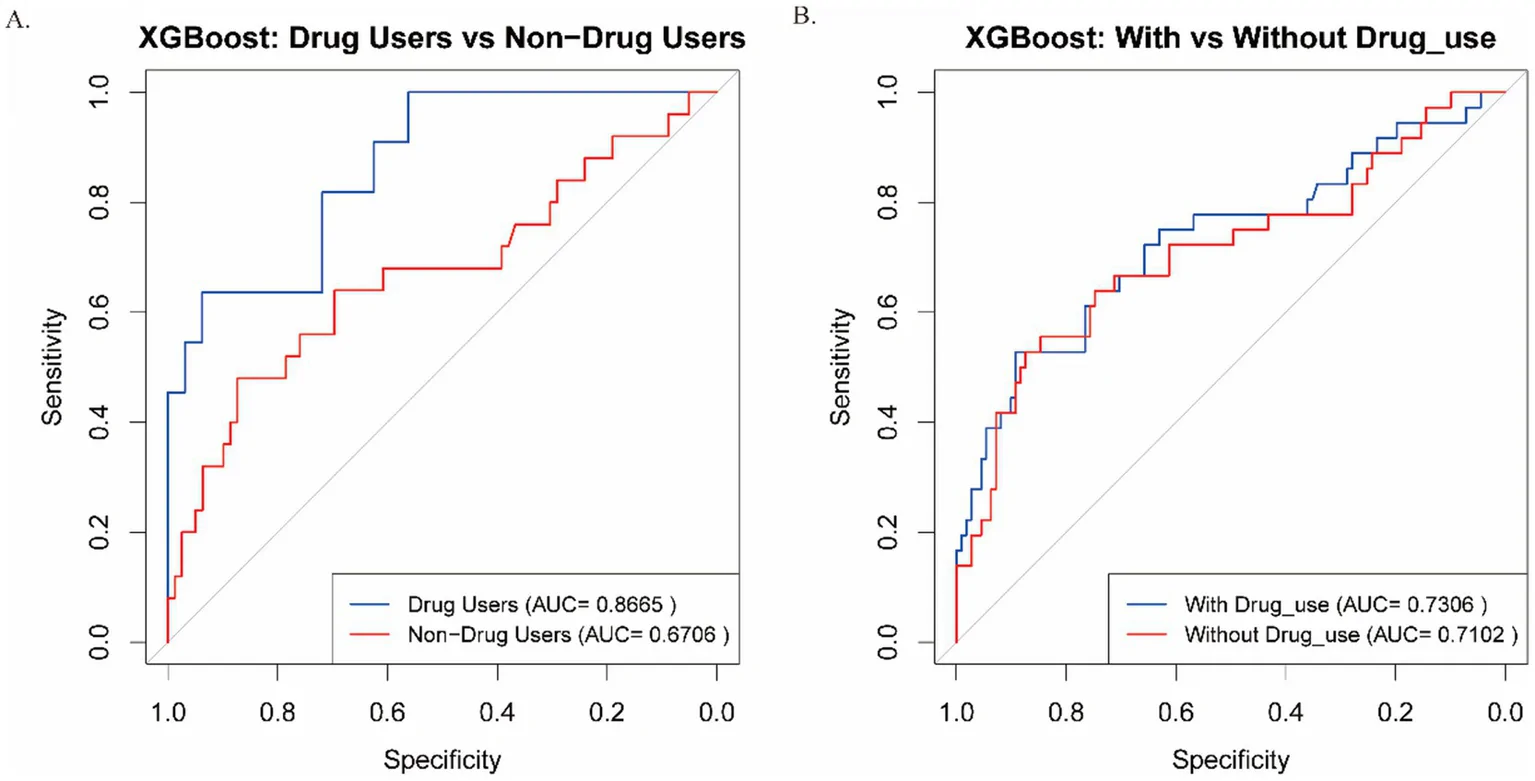
Performance of the XGBoost model in subgroup analysis and feature impact evaluation. **(A)** Receiver operating characteristic (ROC) curves of the XGBoost model in drug-using patients (blue) and non-drug-using patients (red). **(B)** ROC curves of the XGBoost model with the drug_use feature (blue) and without the drug_use feature (red).

### Model explanation

To interpret the prediction mechanism of the optimal XGBoost model and quantify the contribution of each feature, SHAP analysis was performed. The SHAP summary plot revealed that the top 8 most important features for cirrhosis prediction included Platelet, Total bilirubin, ALT, CD8^+^ T cell, Hemoglobin, Adenosine deaminase, CD3^+^ T cell percentage, and AST. Among them, Platelet exhibited the widest range of SHAP values, indicating the strongest discriminative power for identifying cirrhosis, followed by Total bilirubin and ALT. Immune-related indicators such as CD8^+^ T cell and CD3^+^ T cell percentage also contributed substantially, highlighting the critical role of peripheral immune status in HCV-associated cirrhosis (Figure 5A). Notably, hematologic parameters (Platelet, Hemoglobin) and markers of hepatocellular injury (ALT, AST, Total bilirubin) dominated the feature ranking, underscoring that the model relied primarily on liver dysfunction and portal hypertension-related alterations rather than drug-use status. SHAP dependency analysis further revealed that the effects of these key features on cirrhosis prediction were predominantly nonlinear and threshold-dependent, with the direction and magnitude of their impact varying across the feature-value spectrum; such patterns were most evident for Platelet, Total bilirubin, and ALT, whose contributions changed abruptly around specific clinical thresholds (Figure 5B).

**Figure 5:**
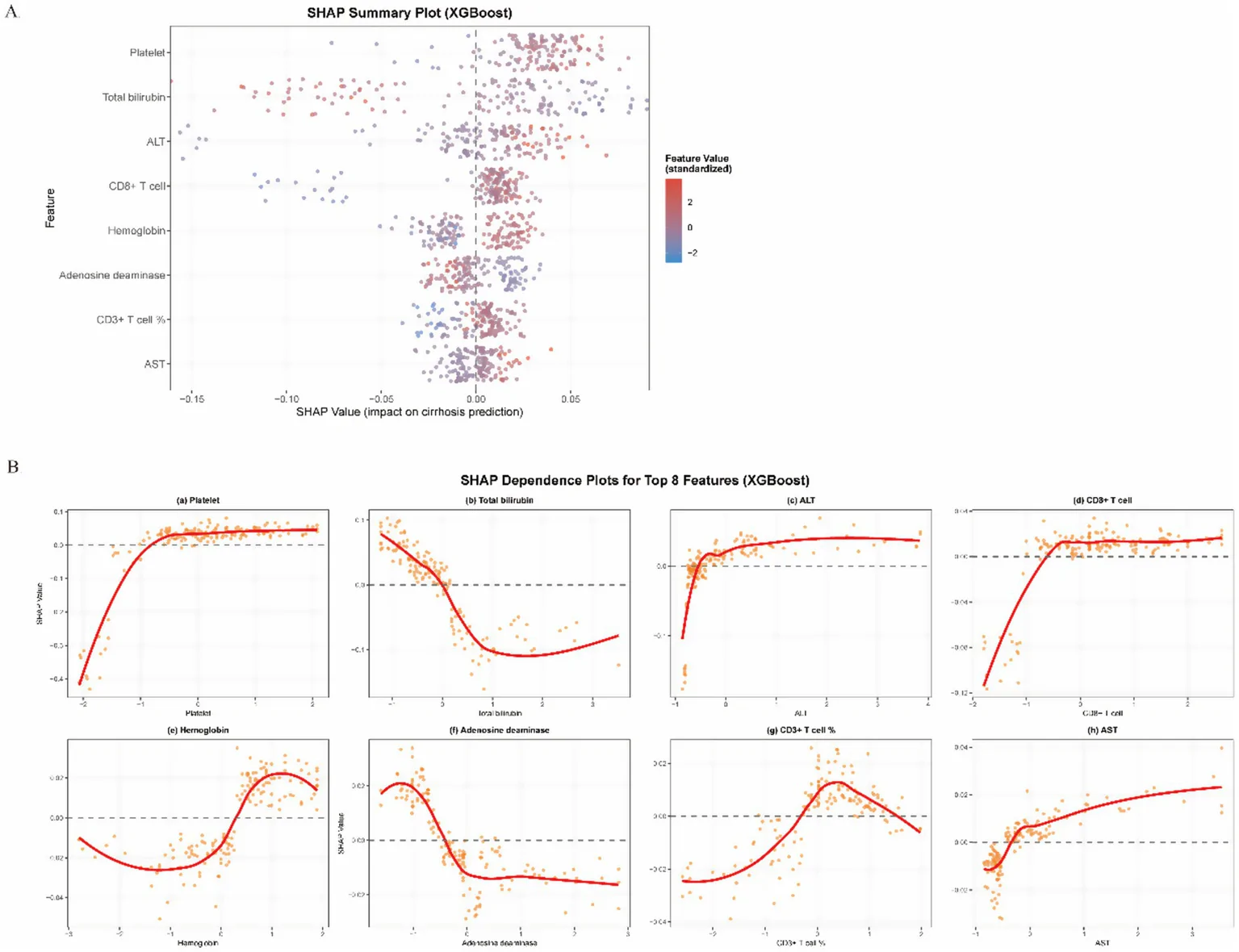
SHAP analysis of the XGBoost model. **(A)** SHAP summary plot of the top 8 features, ranked by contribution to cirrhosis prediction. Color intensity represents feature value. **(B)** SHAP dependence plots of the top 8 features. Each subplot illustrates the relationship between a feature’s value and its SHAP value, with the curve representing a LOESS-smoothed trend.

DCA was conducted to evaluate the clinical net benefit of all models. DCA across the seven models demonstrated that within a wide range of threshold probabilities, the ensemble learning models, particularly XGBoost, RF, and LightGBM, achieved higher net benefit than the “Treat All” scheme, the “Treat None” scheme, and the traditional models. Notably, the XGBoost model provided the highest net benefit in most threshold intervals, indicating superior clinical utility, consistent with its optimal discriminative performance observed in the ROC analysis. Conventional models, including Ridge, SVM, and KNN, showed limited net benefit and lower clinical applicability across most threshold ranges (Figure 6A). Stratified DCA of the XGBoost model revealed that the drug-using subgroup achieved substantially higher clinical net benefit than the non-drug-using subgroup across nearly the entire threshold range (0–0.75). The XGBoost model consistently outperformed both “Treat All” and “Treat None” strategies in drug users, supporting its superior performance and translational potential for cirrhosis risk stratification in this high-risk population (Figure 6B).

**Figure 6:**
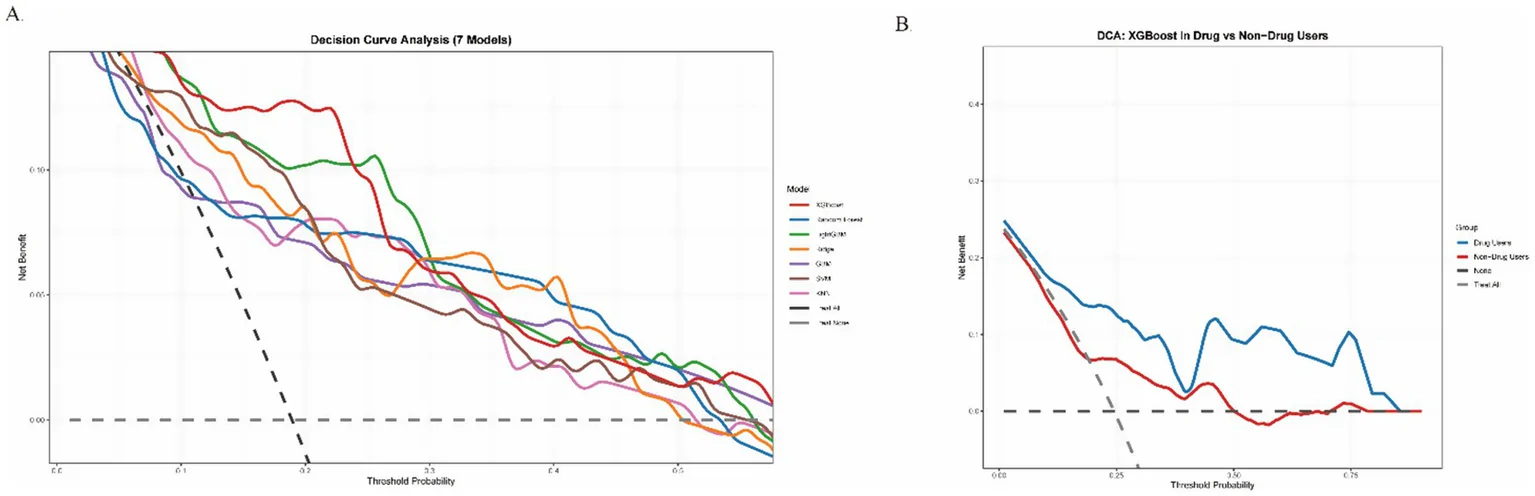
Decision curve analysis (DCA) of machine learning models. **(A)** Net benefit of seven machine learning models across different threshold probabilities. **(B)** Stratified DCA of the XGBoost model in drug-using and non-drug-using subgroups.

## Discussion

Based on drug exposure status and hepatic disease progression stage, patients with HCV infection were divided into four groups: drug users with chronic hepatitis C, drug users with liver cirrhosis, non-drug users with chronic hepatitis C, and non-drug users with liver cirrhosis, followed by comprehensive baseline comparison. Demographic and laboratory analyses revealed significant inter-group differences in age distribution and gender composition. In terms of lymphocyte subsets, CD3^+^ total T cells, CD3^+^ T cell percentage, CD45^+^ lymphocyte count, and CD8^+^ T cell level differed significantly between subgroups. For liver function biochemical indicators, ADA and CHE also exhibited remarkable inter-group discrepancies (22). Such inter-group heterogeneity of immune and liver function indicators confirmed that the progression of HCV infection to cirrhosis is accompanied by obvious peripheral immune disturbance and abnormal hepatocellular synthetic and injury function. Drug-using patients exhibited more pronounced immune imbalance and hepatic biochemical abnormalities, which may have contributed to the observed inter-group heterogeneity and facilitated model discrimination.

The progression of HCV-related cirrhosis is characterized by persistent intrahepatic inflammation, disrupted immune homeostasis and abnormal hepatocellular metabolism. Complex nonlinear correlations and potential interactions exist among clinical indicators. Thus, single biomarkers and traditional regression models are insufficient to fit the complicated disease evolution patterns. Seven machine learning algorithms were compared in this study. The results demonstrated that ensemble learning models including XGBoost, Random Forest and LightGBM exhibited better discriminative performance than traditional models such as Ridge, SVM, and KNN, which was consistent with previous machine learning studies on liver diseases (25, 26). As an optimal algorithm, XGBoost effectively reduced overfitting through gradient boosting and built-in regularization strategies, maintaining stable performance in both cross-validation and independent test cohorts. Furthermore, it enabled the quantification of feature importance and marginal effect trends, satisfying the requirements of transparency and biological interpretability in clinical research.

The SHAP analysis quantifies the predictive contribution of each feature to the model rather than establishing biological mechanism. The top 8 predictive features screened by the SHAP interpretability framework were mainly categorized into peripheral lymphocyte subsets, hepatocellular injury markers, and markers of hepatic dysfunction and portal hypertension. Among the immune parameters, CD8^+^ T cells and CD3^+^ T cell percentage served as core components regulating antiviral immune response and the intrahepatic inflammatory microenvironment (27). Persistent HCV infection induces peripheral T lymphocyte subset imbalance. Abnormal activation of CD8^+^ effector T cells exacerbates hepatic inflammatory infiltration, thereby facilitating hepatic stellate cell activation and liver fibrosis progression (28). For liver function indicators, adenosine deaminase is highly sensitive to chronic intrahepatic immune activation and can early reflect subclinical hepatocellular injury, presenting great value in non-invasive screening of compensated cirrhosis (29). Aspartate aminotransferase is primarily located in hepatocellular mitochondria, and its elevation indicates severe hepatocellular injury and necrosis, objectively reflecting the pathological damage of liver tissues (30). Most of the top 8 predictive features largely overlapped with indicators showing significant inter-group differences, although exceptions (ALT, AST) existed, underscoring the value of multivariate joint discrimination (31). In addition, total bilirubin reflected the excretory dysfunction of hepatocytes, while platelet count and hemoglobin, which are closely related to portal hypertension and hypersplenism, captured the hemodynamic consequences of advanced fibrosis. Notably, most of the top-ranked features, such as ADA, platelet, and CD8^+^ T cells, coincided with the indicators showing significant inter-group differences (32, 33). However, some markers (e.g., ALT and AST) contributed to the model despite lacking significant between-group differences in univariate analysis, underscoring the added value of multivariate joint discrimination for cirrhosis identification.

Subgroup stratified analysis between drug users and non-drug users yielded clinically meaningful results: the XGBoost model achieved a significantly higher predictive AUC in the drug-using subgroup than in the non-drug-using subgroup, indicating distinct population-specific predictive superiority. From the pathophysiological perspective, HCV-infected drug users usually sustain persistent systemic immune activation and chronic inflammatory status. These patients exhibit more severe hepatic parenchymal injury. In addition, they have obvious lymphocyte subset disturbance and abnormal liver biochemistry. Such prominent phenotypic differences between hepatitis and cirrhosis subgroups can be efficiently captured by machine learning algorithms. Decision curve analysis further validated that the XGBoost model maintained stable net clinical benefit across a wide range of threshold probabilities, with more prominent advantages in high-risk drug-using populations. From a clinical translation perspective, the proposed model could serve as a non-invasive pre-screening tool to prioritize patients for confirmatory diagnostic workup. For patients with a model predicted probability of cirrhosis exceeding the clinically chosen threshold, the expected downstream management would include further diagnostic evaluation—such as abdominal ultrasonography or computed tomography, liver elastography when available, and referral to a hepatology specialist—to confirm the presence of cirrhosis and to assess complications including esophageal varices and hepatocellular carcinoma. For patients below the threshold, periodic routine follow up would be appropriate, with repeat assessment if clinical status changes. The decision curve analysis, which was based on the independent test-set predicted probabilities, demonstrated that the XGBoost model provided higher net benefit than both the “Treat All” and “Treat None” strategies across a wide range of thresholds, supporting its clinical utility in guiding these referral decisions. This model is particularly valuable in resource-limited settings and in high-risk populations such as drug users, where access to specialized hepatology care and elastography may be limited. Moreover, some markers used for modeling, particularly CD3^+^/CD8^+^ T-cell subsets, which require flow cytometry, and adenosine deaminase (ADA), may not be routinely available in all HCV screening settings, especially in primary-care or resource-limited facilities. From a clinical perspective, these assays are generally accessible in tertiary hepatology centers and infectious-disease hospitals, the setting from which our cohort was derived, but may be absent in community-based screening programs. Statistically, the inclusion of these immune markers contributed materially to model performance: the SHAP analysis identified CD8^+^ T cells and CD3^+^ T-cell percentage among the top 8 predictive features, and their removal would likely reduce discriminative ability, as reflected by the lower AUC of simpler scores such as FIB-4 (0.79) and APRI (0.73) that rely exclusively on conventional liver-function and blood-count parameters. From a clinical perspective, these assays are generally accessible in tertiary hepatology centers and infectious-disease hospitals, the setting from which our cohort was derived, but may be absent in community-based screening programs. Statistically, the inclusion of these immune markers contributed materially to model performance: the SHAP analysis identified CD8^+^ T cells and CD3^+^ T-cell percentage among the top 8 predictive features, and their removal would likely reduce discriminative ability, as reflected by the lower AUC of simpler scores such as FIB-4 (0.79) and APRI (0.73) that rely exclusively on conventional liver-function and blood-count parameters.

Several limitations of this study should be acknowledged. First, this was a single-center retrospective cohort study, which inevitably incurred selection bias and data collection bias. Further external validation based on multi-center prospective cohorts is required to evaluate the generalization and robustness of the model. Second, only routine hematological indicators including demographics, liver function and lymphocyte subsets were enrolled, without integrating imaging characteristics, fibrosis stage and behavioral data, leaving room for further performance optimization. Third, drug exposure information relied mainly on self-report, and potential underreporting might cause grouping bias. Furthermore, due to the protection of patient privacy and the limitations of routine clinical records, we are unable to further distinguish the patients’ medication usage, including the injection route and non-injection route, the specific types of substances involved, or the current versus previous usage patterns. The exclusion of patients with prior antiviral therapy, HIV co-infection, and major comorbidities necessarily restricts the applicability of our findings to contemporary HCV populations, particularly drug users. In the era of direct-acting antivirals, a substantial proportion of HCV-infected individuals, including many drug users, have already received antiviral therapy, and HIV co-infection and comorbidities are more prevalent in this population. Our model is therefore best positioned as an initial risk-stratification tool for untreated, monoinfected HCV patients without major comorbidities, and it should be validated and, if necessary, recalibrated in broader real-world cohorts that include treated patients, HIV-coinfected individuals, and those with comorbidities. Fourth, the D-Cirr subgroup comprised only 39 cases, reflecting the lower clinical encounter rate of patients with advanced cirrhosis attributable to drug use in real-world settings (34). Although we strengthened regularization and adopted stratified cross-validation to mitigate overfitting, the small sample size may limit statistical power for detecting subgroup-specific effects. In addition, because cirrhosis status in the medical records was recorded as a binary diagnosis without systematic classification into compensated versus decompensated stages, we were unable to report the numbers of compensated and decompensated cirrhosis cases, which precluded stage-specific evaluation of the model across different degrees of hepatic functional reserve. We therefore suggest that decompensation status be captured prospectively in future multicenter studies to enable stage-specific assessment. Future studies with larger, multicenter cohorts are needed to externally validate these findings. Finally, this study only focused on cross-sectional prediction of cirrhosis risk, and long-term follow-up is needed to explore the predictive value for cirrhotic complications and long-term survival outcomes.

In conclusion, based on baseline data of four HCV-infected subgroups, the XGBoost model was identified as the optimal predictive model by comparing seven machine learning algorithms. The top 8 core immune and liver function features were determined via SHAP interpretability analysis, confirming that indicators with significant inter-group differences are pivotal biological markers for identifying HCV-related cirrhosis. Stratified analysis verified that the model achieved superior predictive performance in drug-using populations. This finding well matched their characteristics, including more typical liver injury phenotype, limited screening accessibility, and prominent privacy concerns. Feature ablation indicated that drug_use was not an independent predictor in the model, as its removal did not significantly alter overall AUROC. However, this associational finding does not exclude an independent association between drug use and cirrhosis, and formal mediation analysis would be required to test causal pathways. This model is constructed on routine clinical indicators, giving it favorable interpretability and high adaptability to high-risk populations. It can thus provide a reliable non-invasive basis for early screening, risk stratification, and standardized clinical management of cirrhosis in general HCV-infected individuals as well as in high-risk drug-using populations.

## Data Availability

The raw data supporting the conclusions of this article will be made available by the authors, without undue reservation.
